# Feelings of Parental Authenticity Moderate Concurrent Links Between Breastfeeding Experience and Symptoms of Postpartum Depression

**DOI:** 10.3389/fgwh.2021.651244

**Published:** 2021-05-05

**Authors:** Mikayela Hammond, Rebecca J. Brooker, Sejal Mistry-Patel, Rebecca J. Schlegel, Matthew Vess, Maria Wines, Jessica Havens

**Affiliations:** ^1^Department of Psychological and Brain Sciences, Texas A & M University, College Station, TX, United States; ^2^College of Nursing, Montana State University, Bozeman, MT, United States

**Keywords:** authenticity, breastfeeding, postpartum depression, mother, parenting

## Abstract

A negative breastfeeding experience is a contextual risk factor for the development of postpartum depressive symptoms among mothers. Many current interventions targeted at disrupting this association rely on the ability to make breastfeeding experiences positive. As a beginning step toward identifying alternative approaches, we investigated a potential psychological buffer of the negative relation between breastfeeding experience and symptoms of postpartum depression: feeling authentic in one's role as a parent. Authenticity appears to enhance well-being and buffer negative outcomes more generally, but has largely gone unaddressed in mothers, particularly during the critical peripartum period when depressive symptoms are at increased prevalence. We tested whether three facets of felt authenticity in the parent role (authentic living, acceptance of external influence, and self-alienation) moderated the association between satisfaction with breastfeeding experience and postpartum depressive symptoms in mothers (*N* = 92, 81% White, 85% non-Hispanic, college-educated, *M*_age_ = 30.49). We found that mothers who felt high in authentic living in the parent role showed fewer depressive symptoms when breastfeeding experiences were positive. In addition, we found that the association between negative breastfeeding experience and greater postpartum depression was mitigated when feelings of self-alienation in the parent role, or the sense that one is unaware of or disconnected from who “she really is” as a mother, were low. This work suggests that enhancing women's feelings of connectedness to “who they truly are” as a mother may be protective against some of the negative mental health effects linked to problems with breastfeeding.

## Introduction

Roughly 10% of postpartum women in the United States suffer from major depressive or dysthymic disorders (1). Postnatal stressors, including dissatisfaction with the infant feeding experience, comprise large and stable risk factors for maternal postnatal depression. Dissatisfaction with infant feeding, in particular, is linked to a more than doubled risk for maternal depression in the early postnatal period, even when other stressors are controlled (2). This is especially notable given that an estimated two-thirds of mothers do not meet their own goals for durations of exclusive breastfeeding during the perinatal period (3). A large number of intervention efforts target successful breastfeeding as an outcome (4, 5), in theory reducing the potential for negative outcomes like depression by eliminating problems with breastfeeding. However, because a successful and/or satisfying breastfeeding experience is not feasible for every mother, community and provider-based interventions that do not rely on successful breastfeeding may offer unique protections against postpartum depression. Thus, the identification of additional factors that may protect against postpartum symptoms of depression will be important for advancing both theories of postpartum depression and the design and application of interventions.

The majority of new mothers express an intention to breastfeed (6, 7) and at least one study has shown that more than 80% of babies are indeed breastfed between birth and 3 months (8). This work suggests that most mothers attempt to follow through on their breastfeeding intentions. Nonetheless, rates of exclusive breastfeeding decline sharply in the first 6 months of life which is the period recommended for exclusive breastfeeding by the American Academy of Pediatrics (9).

Accordingly, most mothers report that they stop breastfeeding earlier than they would like (10), which is notable given that a negative breastfeeding experience is linked to increased depressive symptoms (11). Breastfeeding experience is, in some cases, discussed as a distal consequence of maternal depression, but is also a robust *predictor* of depressive symptoms (12). Indeed, dissatisfaction with infant feeding experience uniquely predicts a substantial increase in risk for postpartum depression (13). Mothers who wish to but who are unable to successfully breastfeed are often those with lactation issues, who develop illnesses, or who have concerns about pumping milk or infant nutrition and weight (10). Related to their negative feeding experiences, these mothers may have more negative interactions with their infants, leading to an experience of more negative emotion overall (12). Additionally, changes in hormone levels associated with breastfeeding implicate a possible biological pathway by which breastfeeding ultimately decreases the experience of stress and arousal (12), a protective factor that is absent for mothers unable to breastfeed. Notably, the clearest way to intervene to prevent depressive symptoms per these pathways is to improve the quality of breastfeeding experience.

However, given that it may not always be feasible to directly change every mother's breastfeeding experience, identifying other protective factors that may disrupt the link between negative breastfeeding experience and maternal depression is important. To this end, mothers who feel authentic in their role as a parent may be protected from depressive symptoms during the postnatal period, particularly if such feelings of authenticity buffer the impact of a negative feeding experience. Authenticity has been conceptualized as a subjective experience of “unimpeded operation of one's true- or core self in one's daily enterprise” [(14), p. 344]. A prominent model of authenticity suggests that authenticity includes three facets: authentic living, acceptance of external influences, and self-alienation (15). Conceptually, authentic living reflects the degree to which people feel that their feelings and behavior are congruent with who they think they truly are. Acceptance of external influence reflects the degree to which people let others determine their behavior and experiences. Self-alienation reflects the degree to which people feel disconnected from or unaware of who they “truly are.”

In general, authenticity is positively related to psychological health (16, 17) and resilience in the face of stress and adversity (16, 18). For example, the relation between loneliness and depressive symptoms is moderated by perceived authenticity, such that individuals who feel highly authentic are less impacted by the negative effects of loneliness (19). Though there are differing views on why perceived authenticity may relate to resilience, one idea that is relevant to breastfeeding difficulties is that perceived authenticity may make setbacks seem less burdensome (20). This is consistent with the identity-based motivation model (21, 22), which posits that when a behavior feels identity-consistent, difficulties are more likely to be interpreted as a challenge and opportunity to grow as opposed to signals of one's inability in that domain. Thus, a new mother who experiences challenges with breastfeeding, but feels authentic in her role as a parent, may be better positioned to interpret that difficulty as an opportunity to grow as a mother as opposed to a signal of her inability to be a good parent. Consistent with this perspective, other research outside of the parenting domain has shown that perceived authenticity elicits shame-free guilt (but not guilt-free shame) in response to shortcomings (23). This is important because shame-free guilt is widely considered to be more of an adaptive response to shortcomings in that it divorces negative feelings from more global negative self-evaluations that are characteristic of depression (24, 25). Given the apparent ability of feelings of authenticity to foster resilience in the face of difficulties, it is thus plausible that high levels of authenticity, particularly those related to being a parent, may similarly buffer the association between negative breastfeeding and maternal depressive symptoms.

While authenticity in relationships has been linked to the mitigation of depressive symptoms of adolescence (26), another critical period for the development for depression, the role of authenticity in the manifestation of maternal postpartum symptoms of depression has gone unaddressed. The existence of such a pathway would identify an important potential strategy for psychological intervention that does not rely on breastfeeding success. Thus, the aim of the current study was to test the possibility that feelings of authenticity can modulate the link between negative breastfeeding experiences and postpartum depressive symptoms. We hypothesized that the negative association between breastfeeding experiences and depressive symptoms would be mitigated when levels of parent authenticity were high. We tested this effect using an existing longitudinal data set that allowed us to control for levels of prenatal depression symptoms in mothers, prior to the initiation of breastfeeding.

## Materials and Methods

### Participants

Participants included women enrolled in a larger longitudinal study of emotional development that involved two laboratory visits during pregnancy (second trimester, third trimester) and one postpartum (27, 28). Ninety pregnant women in their second or third trimester were targeted for participation; target sample size was derived from power analyses conducted for the main hypothesis of the parent study. In order to enroll in the study, women needed to be in their second or third trimester of pregnancy at the time of recruitment, not taking any stimulant medications, and free of any known neurological impairments. Ninety-four pregnant women responded to advertisements for the study. Because we anticipated attrition over time and no risk is associated with additional power, we enrolled all 94 women in the study. Rolling recruitment procedures resulted in 81 participants at the second trimester visit, 85 mothers at the third trimester visit, and 75 mothers at the postnatal visit. Women were recruited through informational brochures at local doctors' offices (26%), advertisements at the local University (25%), word-of-mouth referrals (27%), flyers at local preschools and businesses (13%), referrals from the local Women, Infants, and Children office (7%), or announcements from local mothers' clubs (2%).

Participant demographics are given in Table 1. Consistent with the sociodemographics of the population from which the sample was drawn, participants (who chose to report race and ethnicity) were mostly White and non-Hispanic (Table 1). The median and mode level of education was a college degree (36%). Gross annual family income ranged from <$15,000 to more than $91,000; the modal reported earnings were $51,000-$60,000 or more than $91,000 per year. On average, mothers were in their early thirties at the time of the second trimester visit, though maternal age ranged from 21 to 41 years (*M* = 30.49, SD = 4.22). Sample mean characteristics thus largely represent White, non-Hispanic, middle-class mothers.

**Table 1:** Participant demographics (*N* = 92).

**Variable**	***n***	**Percentage**
**Education**		
High school graduate	4	4.3
Some college	18	19.6
4-year college degree	37	40.2
Advanced degree	33	35.9
**Race**		
White	81	88.0
Asian	7	7.6
American Indian-Alaska native	1	1.1
Black	1	1.1
Mixed race	1	1.1
Did not report	1	1.1
**Ethnicity**		
Hispanic or Latino	2	2.2
Not Hispanic or Latino	85	92.4
Did not report	5	5.4
**Income**		
$15,000 or less	7	7.6
$16,000 to $20,000	3	3.3
$21,000 to $30,000	6	6.5
$31,000 to $40,000	9	9.8
$41,000 to $50,000	8	8.7
$51,000 to $60,000	14	15.2
$61,000 to $70,000	5	5.4
$71,000 to $80,000	3	3.3
$81,000 to $90,000	5	5.4
$91,000 or more	21	22.8
Did not report	11	12.0
**Feeding**		
Breastfeeding only	32	34.8
Supplementing breastfeeding with bottle	23	25.0
Bottle feeding only	13	14.1
Did not report	24	26.1

Participants included both primiparous and multiparous mothers. Rates of pregnancy and labor and delivery complications, as reported postnatally, were low (*M* = 3.35, SD = 2.22). The most commonly reported pregnancy complication was a fever or chills (*n*= 7), and the most common delivery-related risk factor was labor induction (*n* = 21). Participants also reported using very few medications during pregnancy (*M* = 1.02, SD = 1.13), most frequently reporting over-the-counter treatments for heartburn or nausea. The majority of infants were born full-term (*M* = 39.64, SD = 1.75 weeks). Only six infants were identified as being born prematurely.

All procedures were approved by the Human Subjects Committee of the Institutional Review Board at Montana State University (RB011615-FC). At each assessment, a research assistant provided mothers with a written consent form and verbally described study procedures. Mothers signed consent forms before participating. Participation in the parent study included two prenatal laboratory visits: one each during the second (*M* = 21.15 weeks; SD = 3.79) and third trimester (*M* = 35.92 weeks; SD = 1.47), and a final visit at 4 months postpartum (*M* = 4.27, SD = 0.62). The timing of assessments was selected based on the goals of the parent study and were intended to capture changes in maternal mental health from pre to postpartum within a period of available funding. Prenatal assessments were scheduled over the phone with a research assistant or via an online booking site. Participants scheduled their postnatal assessment during their prenatal appointment. Because the postnatal visit targeted infants at 4 months of age, laboratory staff confirmed the child's birth date roughly 1 month after the anticipated due date. This visit to the laboratory also included physiological data collection, maternal responses to previous visualizations, and a standardized infant temperament battery, none of which are used in the current report.

Each wave of data collection included psychophysiological, behavioral, and survey assessments. Given our interest in postpartum changes in depressive symptoms, the current report focuses on postpartum assessments of parent characteristics, experience and mental health collected at infant age 4 months postpartum (i.e., postnatal assessment) while controlling for maternal mental health at the third trimester assessment (i.e., prenatal assessment).

### Measures

#### Breastfeeding Experience

Mothers' experiences with breastfeeding were assessed via a single, face-valid self-report item that asked mothers to rate, on a 10-point scale, their overall experience with feeding their infant (1 = terrible, 10 = terrific). This scale resembles that used by Granberg et al. (29). Additional information on the validation of this item is available in the supplementary material.

#### Authenticity in the Parent Role

Authenticity in the parent role was assessed at the postnatal assessment. Mothers completed a survey to assess three domains of authenticity, or the feeling of knowing and expressing who one truly is, specific to their role as a parent. Mothers were also asked about domains of authenticity relative to their role as spouse/partner; however, this work focused on the parent role given our hypotheses about breastfeeding. Each facet of authenticity was assessed in accordance with traditional definitions: authentic living (the degree to one feels that his/her feelings and behavior are congruent with who s/he truly is), acceptance of external influence (the degree to which one lets others determine their behavior and experiences), and self-alienation (the degree to which one feels disconnected from or unaware of who they “truly are”). The measure was identical to that developed by Wood et al. (15) except that our instructions asked mothers to think specifically about themselves in the parent role and indicate the degree to which statements were true for them (1 = not at all, 7 = extremely). This approach follows precedent for measuring authenticity within specific social roles (30). Two statements assessed each domain of authenticity. Items were mean composited to create final scores reflecting authentic living (α = 0.747), accepting external influence (α = 0.741), and self-alienation (α = 0.726) in the parent role.

#### Symptoms of Depression

Levels of depression during the postpartum period were measured using the Beck Depression Inventory (31) and the Edinburgh Postnatal Depression Scale (32). The BDI included 20 items that asked mothers to rate the degree to which they experienced, in the last week, numerous symptoms of depression. Ratings across all items were summed such that higher values indicated greater levels of depression. Internal consistency on the BDI was acceptable (α < = 0.844) and mothers reported levels of symptoms ranging from normal range (0–9; 51.6%) to extremely severe depression (30–60; 1.1%). We note that the original BDI includes 21 items, but the item assessing suicidality was removed for this study. The EPDS included 10 items that asked mothers to rate the degree to which statements reflecting symptoms of depression were true for them over the past week (0 = never, 3 = most of the time). Internal consistency for the EPDS was acceptable (α = 0.821). A portion of mothers in the sample (≥10; 11.9%) had scores that indicated the possible presence of postpartum depression. BDI and EPDS scores were highly correlated, suggesting that they reflected assessed the same construct (*r* = 0.817). Given that the scales for each measure were different, scores were standardized (transformed to z-scores) and mean composited to form a single score indicating level of postpartum depression.

#### Covariates

Given evidence that, in many cases, the onset of depressive symptoms that result in a postpartum diagnosis actually begin prenatally (33), we also included maternal depression scores from the prenatal assessment. This enabled us to statistically control for levels of depression that may have been present before mothers were trying to breastfeed their infants and isolate emerging symptoms of postpartum depression as our outcome. Prenatal depression scores were calculated in a manner identical to scores for postnatal depression, using BDI (α = 0.869) and EPDS (α = 0.876) ratings from the third trimester visit.

In addition, in order to ensure that results were not influenced by general levels of authenticity, we isolated maternal feelings of authenticity specific to the parent role by also including maternal ratings on each facet of authenticity that were general, or non-specific to the parenting role (15). Definitions for general authenticity and each facet of general authenticity were identical to those used for the measure of authenticity in the parent role. Participants were instructed to report the degree to which each of 12 items was true for them (1 = not at all true of me; 7 = very true of me). Four items were mean composited to create scales for authentic living (α = 0.697), accepting external influence (α = 0.835), and self-alienation (α = 0.756).

#### Missing Data

Two mothers did not participate at any time point after enrolling in the study. Sixty-five mothers provided data across all three assessments and nearly all mothers (91%) provided data at multiple assessments (two out of three). Specific to our measures of interest, twenty mothers were missing postnatal depression scores, 22 mothers were missing reports of breastfeeding experience, and 19 mothers were missing reports of self-alienation in the parent role. Minimum covariance coverage was 0.701. Independent-samples *t*-tests suggested that mothers who were missing postnatal depression scores [*t*(80) = −2.701, *p* = 0.008] and mothers missing ratings of authenticity in the parent role [*t*(80) = −2.244, *p* = 0.028] reported higher levels of prenatal depression. No other patterns of missing data were observed. Thus data were assumed to be missing at random (MAR). Missing data were handled using a full-information maximum likelihood (FIML) procedure that uses all available data to estimate non-biased parameter estimates, thus allowing one to take advantage of the full sample from which at least some data are available. FIML is appropriate with MAR data when variables that can account for patterns of missingness are included in the analysis (34). However, because FIML relies on patterns of available data, we did not include mothers who were missing data for all of the measures in the current study. Thus, the final analytic sample comprised 87 mothers. All measures and the final data set that support the conclusions of this article is available on the Open Science Framework https://osf.io/ct42e/?view_only=9543558c86514b8f979f828083ae558d.

#### Plan for Analysis

Multiple regression analyses were conducted in Mplus 7 Version 1.4 (35). Total postpartum depressive symptoms were regressed onto facets of authenticity in the parent role (authentic living, acceptance of external influences, and self-alienation, tested separately), maternal reports of satisfaction with the breastfeeding experience, and the interaction between self-alienation and breastfeeding experience. Because we were not exclusively interested in non-overlapping variance, each facet of authenticity was tested in a separate model. Multiple regression models were presumed to fit standard assumptions of linearity, homoscedasticity, independence, and univariate normality. The FIML imputation technique additionally assumes that residuals are normally distributed.

Prenatal depression was included as a covariate in order to isolate effects of predictors on symptoms of postnatal depression, consistent with study aims. Similarly, ratings for authenticity that were non-specific to the parenting role (i.e., general level of authenticity) were also controlled, as was the interaction between these ratings and the breastfeeding experience (36, 37). All variables were centered prior to the creation of interaction terms. Results were considered to be statistically significant if two-tailed probability values were <0.05 and 95% confidence intervals did not include zero.

## Results

Bivariate Pearson correlations (two-tailed; Table 2) suggested that overlap across domains of authenticity was moderate, indicating specificity rather than redundancy for different scales. Postpartum depression symptoms were associated with all of the covariates at the bivariate level, underscoring the need for their inclusion in the regression models. More central to study hypotheses, postpartum symptoms of depression were associated with a worse breastfeeding experience and greater feelings of self-alienation in the parent role. Prenatal symptoms of depression were also positively associated with self-alienation in the parent role during the postpartum period.

**Table 2:** Descriptive statistics and bivariate correlations for primary variables and covariates.

	***M* (SE)**	**1**	**2**	**3**	**4**	**5**	**6**	**7**	**8**
1. Prenatal depression symptoms (*z*-score)	0.00 (0.10)								
2. Authentic living	6.07 (0.09)	−0.06							
3. External influences	3.34 (0.15)	0.15	−0.2						
4. Self-alienation	1.88 (0.10)	0.25	−0.44^**^	0.19					
5. Postpartum depression symptoms (*z*-score)	0.00 (0.12)	0.60^**^	−0.50^**^	0.25^*^	0.49^**^				
6. Authentic living in parent role	6.49 (0.07)	−0.25	0.28^*^	−0.28^*^	−0.24^*^	−0.18			
7. External influences on parent role	2.64 (0.14)	0.12	−0.09	0.26^*^	0.10	0.03	−0.14		
8. Self-alienation in parent role	1.59 (0.11)	0.42^**^	−0.12	0.02	0.15	0.25^*^	−0.19	0.06	
9. Satisfaction with breastfeeding experience	8.51 (0.23)	−0.11	−0.13	0.08	−0.06	−0.26^*^	0.14	−0.06	−0.34^*^

*N = 87*.

* *p < 0.05*,

** *p < 0.001*.

Bivariate correlations and all of the regression models (Table 3) suggested that a more satisfying breastfeeding experience was related to fewer symptoms of postpartum depression (Table 3). In the model that included acceptance of external influences, acceptance of external influences in the parenting role was not associated with symptoms of postpartum depression. More central to the study hypotheses, the interaction between breastfeeding experience and accepting external influence in the parenting role was non-significant. Rather, only main effects were present: greater levels of prenatal symptoms of depression, more negative breastfeeding experience, and greater levels of acceptance of external influences in general were predictive of postnatal symptoms of depression.

**Table 3:** Results of regression analyses predicting maternal postpartum depressive symptoms from facets of authenticity and breastfeeding experience.

**Authentic Living**	***B***	**SE(*B*)**	**95% CI(*B*)**	**β**	***P***
Prenatal depression	0.58	0.14	[0.30, 0.86]	0.50	<0.01
Authentic living	−0.89	0.14	[−1.16, −0.61]	−0.58	<0.01
Authentic living in parenting role	0.21	0.16	[−0.11, 0.53]	0.12	0.19
Breastfeeding experience	−0.23	0.05	[−0.33, −0.13]	−0.40	<0.01
Authentic living X breastfeeding experience	0.30	0.11	[0.09, 0.51]	0.30	<0.01
Authentic living in parenting role X Breastfeeding experience	−0.24	0.10	[−0.42, −0.06]	−0.23	0.01
**External influence**	***B***	***SE*** **(** ***B*** **)**	**95% CI(** ***B*** **)**	**β**	***P***
Prenatal depression	0.63	0.17	[0.30, 0.90]	0.54	<0.01
External influence	0.20	0.08	[0.03, 0.33]	0.22	0.02
External influence in parenting role	−0.08	0.09	[−0.25, 0.07]	−0.08	0.39
Breastfeeding experience	−0.17	0.06	[−0.28, −0.07]	−0.29	<0.01
External influence X Breastfeeding experience	−0.06	0.04	[−0.14, 0.01]	−0.16	0.13
External Influence in parenting role X Breastfeeding experience	0.06	0.05	[−0.04, 0.14]	0.10	0.27
**Self-alienation**	***B***	***SE*** **(** ***B*** **)**	**95% CI(** ***B*** **)**	**β**	***p***
Prenatal depression	0.60	0.15	[0.30, 0.85]	0.53	<0.01
Self-alienation	0.48	0.10	[0.28, 0.65]	0.40	<0.01
Self-alienation in parenting role	−0.13	0.13	[−0.39, 0.08]	−0.12	0.31
Breastfeeding experience	−0.09	0.05	[−0.19, −0.01]	−0.16	0.07
Self-alienation X Breastfeeding experience	0.03	0.04	[−0.05, 0.10]	0.06	0.46
Self-alienation in parenting role X Breastfeeding experience	−0.14	0.06	[−0.25, −0.04]	−0.21	0.02

*N = 87. X in variable name indicates interaction between listed variables*.

**Figure 1:**
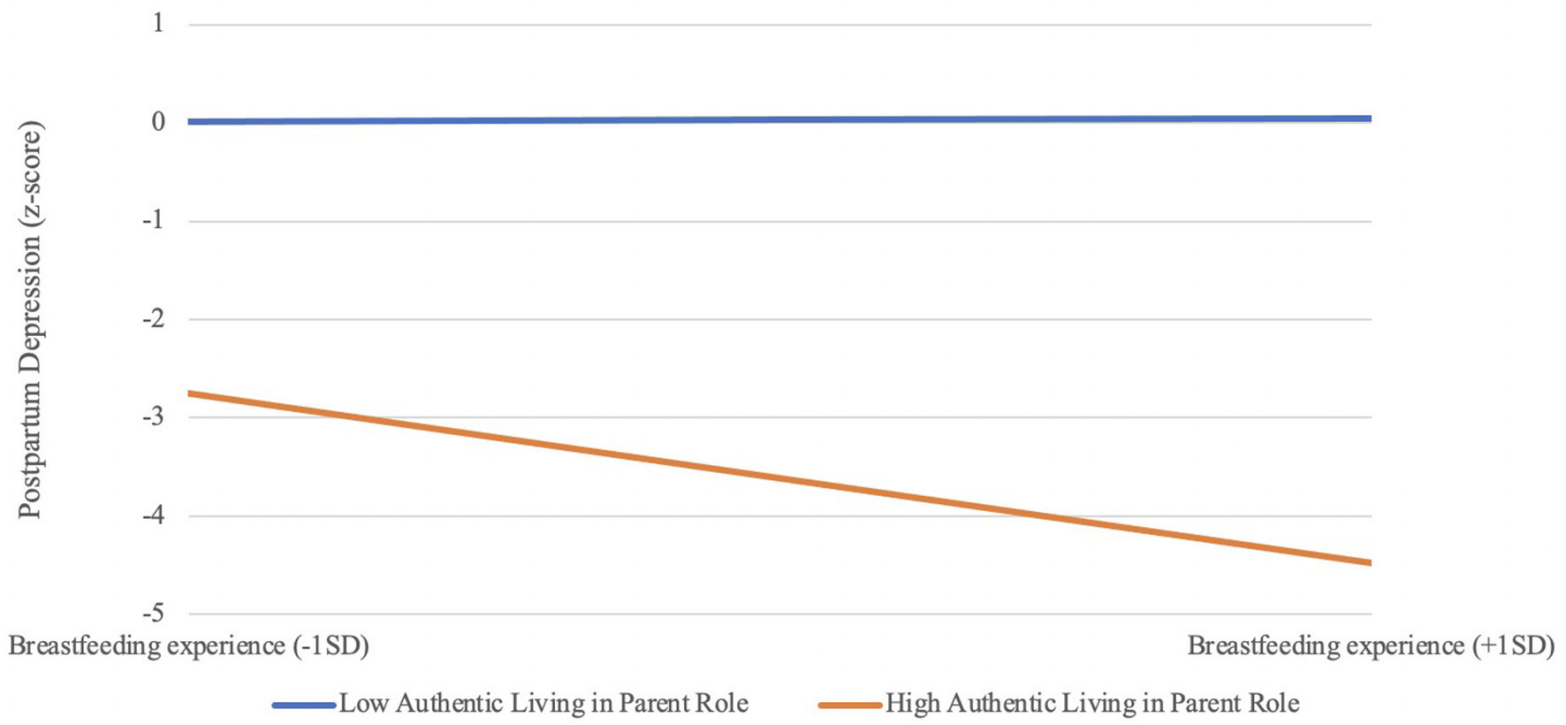
Authentic living in parent role moderates link between breastfeeding experience and symptoms of postpartum depression.

In the model that included authentic living, greater symptoms of prenatal depression, less authentic living, and a worse breastfeeding experience were all associated with higher levels of postpartum depression symptoms. Authentic living in the parenting role was not associated with symptoms of postpartum depression. There was, however, a significant interaction between authentic living in the parenting role and breastfeeding experience predicting symptoms of postpartum depression. A probe of this interaction, per the recommendations of Aiken and West (38), was conducted by recentering the moderator (authentic living in the parenting role) at high (+1 SD) and low (−1 SD) levels and then rerunning the regression model. Probing the interaction in this manner (Figure 1) suggested that breastfeeding experience was unrelated to depressive symptoms at low levels of authentic living in the parenting role (*B* = 0.01, *SE*(*B*) = 0.09, β = 0.02, 95% *CI*(*B*) = [−0.18, 0.19], *p* = 0.92). However, a better breastfeeding experience predicted fewer depressive symptoms (or a more negative experience predicted more symptoms) at high levels of authentic living in the parenting role (*B* = −0.47, *SE*(*B*) = 0.12, β = −0.81, 95% *CI*(*B*) = [−0.70, −0.24], *p* < 0.01). This pattern of findings is consistent with previous results suggesting that a better breastfeeding experience is associated with fewer depressive symptoms, but also suggests that this association is contingent upon feelings that one is behaving in ways that are consistent with their identity as a mother.

**Figure 2:**
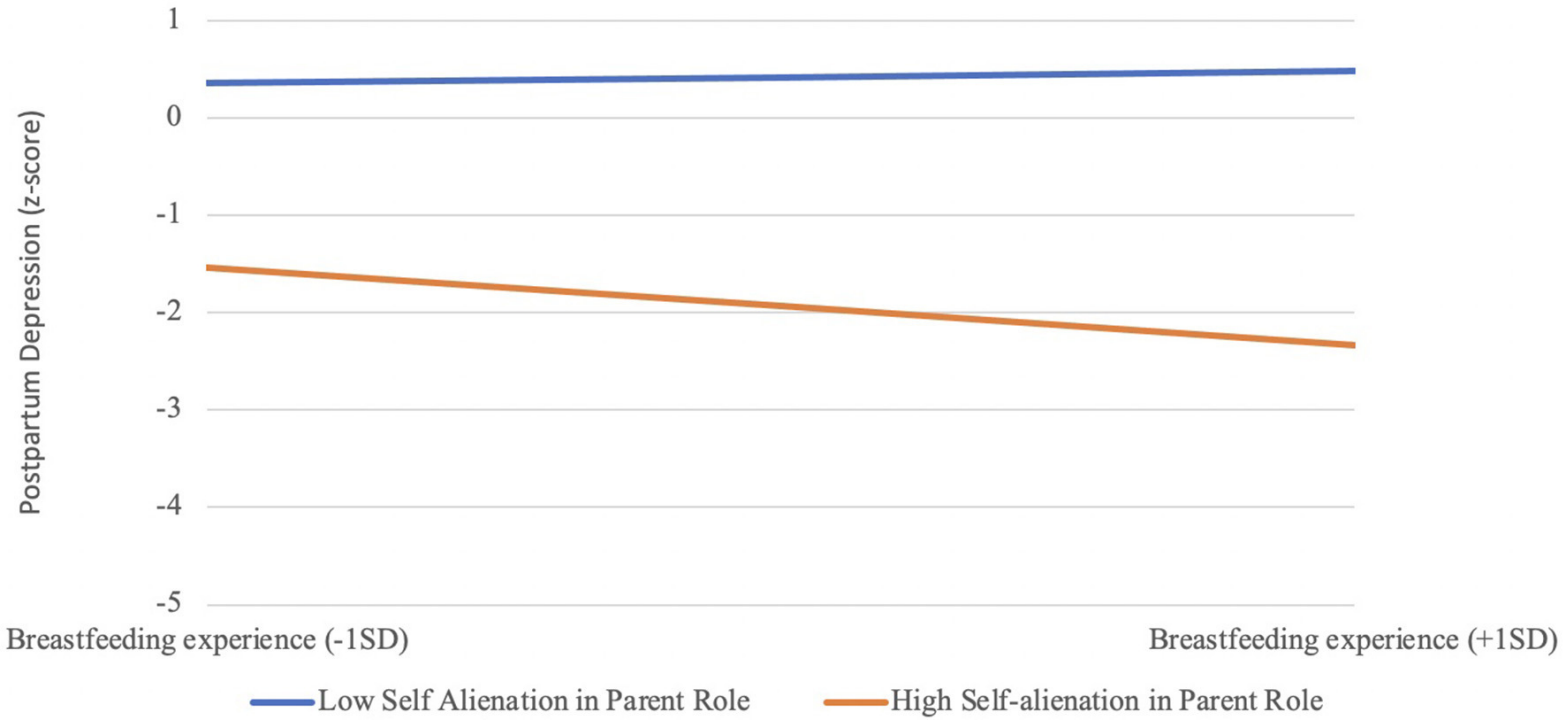
Self-alienation in the parent parent role moderates link between breastfeeding experience and symptoms of postpartum depression.

Greater symptoms of prenatal depression, greater self-alienation, and a worse breastfeeding experience also predicted greater levels of postpartum depression in the model that included self-alienation. Self-alienation in the parenting role did not directly predict symptoms of postpartum depression. However, there was a significant interaction between self-alienation in the parenting role and breastfeeding experience predicting levels of postpartum depression. A probe of this interaction (Figure 2) at high (+1 SD) and low (−1 SD) levels of self-alienation in the parenting role suggested that a less positive breastfeeding experience predicted greater depressive symptoms at high levels of self-alienation in the parent role (*B* = −0.22, *SE*(*B*) = 0.07, β = −0.39, 95% *CI*(*B*) = [−0.35, −0.09], *p* < 0.01), but not at low levels of self-alienation in the parent role (*B* = 0.03, *SE*(*B*) = 0.08, β = 0.06, 95% *CI*(*B*) = −0.12, 0.19], *p* = 0.68). Thus, consistent with hypotheses, the traditional link between negative breastfeeding experience and depressive symptoms appears to be disrupted when mothers are able to maintain a sense of connection to who they “truly are.”

## Discussion

Results were largely consistent with study hypotheses. Authentic living in the parenting role and self-alienation in the parenting role modulated the association between a mother's breastfeeding experience in the infant's first 4 months of life and maternal depressive symptoms. Namely, the traditional link between poorer breastfeeding experiences and greater maternal symptoms of depression (12) was disrupted when mothers were able to maintain a high sense of authentic living and low levels of self-alienation in their role as a parent.

This work adds nuance to an established association between breastfeeding experience and postpartum depression through the lens of authenticity, prioritizing the psychological experience of mothers during interactions with their infants. Specifically, these results suggest that feelings of self-alienation in the parenting role may be an important part of the breastfeeding experience for postnatal mothers. Correlations suggested that satisfaction with the breastfeeding experience was associated with a feeling of not knowing who one is as a mother. However, the interaction pattern suggested that the extent to which a mother can maintain the feeling that she does know who she is as a mother may protect her from the development of depressive symptoms associated with a negative breastfeeding experience. In this way, our work suggests a possible means by which to intervene when mothers are less than satisfied with their breastfeeding experience: breaking the link between dissatisfaction with breastfeeding experience and feelings of self-alienation as a parent. That is, intervention designs might focus on increasing a mother's feeling that she knows who she is even if this one aspect of her experiences with her infant is not going as hoped. The findings also suggest that it is specifically feelings of self-alienation *as a parent* that are important (i.e., more general feelings of self-alienation did not offer the same protection).

Surprisingly, the interaction between breastfeeding experience and authentic living in the parent role appeared to function somewhat differently. In fact, inspection of the recentered means suggests that low levels of authentic living are associated with a greater number of depressive symptoms overall. Thus, it may be that the risk conferred by low authentic living is more difficult to disrupt, even through a positive breastfeeding experience, resulting in the effect of authentic living “overriding” the effects of breastfeeding experience. Instead, we saw that a more negative breastfeeding experience predicted more depressive symptoms when authentic living was high. However, it should also be noted that, as suggested in Table 1, there was little variability in maternal ratings of authentic living in the parenting role which introduces a reduction in range that could bias the estimates of association. Recentering this variable at low levels thus runs the risk of representing very few mothers in the actual data set. This result should therefore be interpreted with caution and should be further investigated in a larger and more variable sample, particularly given that it runs counter to previous findings. This result may reflect a statistical anomaly rather than a meaningful pattern of results.

The bivariate correlations suggested that greater prenatal symptoms of depression were associated with more self-alienation in the parent role during the postnatal period. Because both prenatal symptoms and trait-level self-alienation were included as a covariate in the current analyses, this does not change the interpretation of the current results. Rather, it suggests that there may be a bidirectional association between symptoms of depression and feelings of self-alienation that can compound over time. Additional longitudinal investigations will be important for clarifying such a possibility, with special attention needed for the perinatal period and other critical periods of risk for depressive syndromes [e.g., adolescence; (39)].

The current results offer a basic science foundation for future investigations aimed at designing clinical interventions to prevent postpartum depression. Overall, the findings partially align with broader research on authenticity suggesting that feelings of authenticity may buffer people from the psychological consequences of negative experiences. For example, as noted earlier, research indicates that the experience of loneliness is less consequential for depression among people who have a heightened sense of authenticity (19). Our findings make a similar point, at least in regard to the self-alienation component of authenticity. Negative breastfeeding experiences predicted depression at high levels of self-alienation, but not at low levels of self-alienation. In contrast, our findings for the authentic living component of authenticity yielded a different pattern. Depression was more strongly associated with breastfeeding experiences at high levels of authentic living. As we note, this result was unexpected and they certainly do not align with previous research in the literature (19). Of course, Bryan et al. (19) utilized a global measure of authenticity that did not distinguish between the facets that we isolated in our work. It could very well be the case that a self-alienation component was driving the effects in those studies. At the very least, our findings suggest that it might be important for future work to consider individual facets of authenticity as doing so may highlight more nuanced patterns of findings.

The findings of the current work also highlight the potential importance of clinical interventions that increase a sense of authenticity. In this way, our findings connect to some of the classic, humanistic theorizing of individuals like Rogers (40). Rogers posited that clinical techniques that help align a client's experiences with their core self will ultimately be effective at mitigating psychological dysfunction. This would seem relevant to a discussion of authenticity, as the alignment of experience with one's core sense of self may be central to the subjective experience of authenticity (15, 41). And, indeed, a recent study shows that the aspects of the client-patient relationship emphasized in Rogers' theorizing do, in fact, contribute to an increased sense of authenticity in clients over time (42).

### Limitations and Future Directions

This work is not without limitations. First, the primary variables (self-alienation in the parent role, postpartum depression, and breastfeeding experience) were assessed concurrently. Thus, it is not a design from which we can establish causal associations or long-term effects. Second, it is possible that self-alienation in the parent role plays a mediating, rather than moderating, role in the association between breastfeeding experience and symptoms of postpartum depression. While our sample offers sufficient power for detecting an interaction effect of roughly 0.20 or greater, it is not large enough for a powerful test of mediation unless all effects sizes were to be substantially larger (43), making a null effect difficult to interpret. Thus, this will be an important avenue for future research in a comparable design with a larger sample of mothers. Third, our assessment of breastfeeding experience was a single self-report item. Because our interest is in maternal satisfaction with her own breastfeeding experience, it is necessary to assess via self-report. Similarly, there is evidence that a single, face-valid item can provide as much information as a full questionnaire in adult assessments (44); however, such a possibility has not been tested directly with this item and a multidimensional assessment may reduce error in this measure. Our assessment of facets of authenticity in the parent role was similarly derived from a measure that, although based on a common assessment in the literature, was developed specifically for this sample and study. Finally, our sample included mothers that are largely white and middle class, most of whom lived near a University town. Future work should focus on increasing sample diversity, both in terms of race, ethnicity, and socioeconomic status.

## Conclusions

Despite limitations, this work uses a novel design and sample to demonstrate a putative role of self-alienation in the manifestation of depressive symptoms in postpartum mothers. This work aligns with existing work on the psychological benefits of being aware of and feeling authentic and extends to the domain of breastfeeding. Indeed, examining how role-specific feelings of true self-alienation shape responses to breastfeeding experience highlights the utility of extending work on true self-alienation to specific contexts of vulnerability. As such, it both adds to the extant literature on the importance of authenticity and self-alienation across a spectrum of well-being-related outcomes. Moreover, elucidating this particular pathway offers a novel possible point of intervention for mothers who may be at risk for postpartum depression, offering guidance to intervention scientists and care providers regarding ways to detect risk and intervene early in patient care. Although there are not currently established interventions for individuals who experience high levels of self-alienation, our work adds to the evidence for the possible utility of pursuing such an avenue of research. An intervention that can decrease levels of self-alienation in the parent role may offer protective benefits for mothers who ultimately have a negative experience with breastfeeding.

## Data Availability Statement

The dataset of this article is available on Open Science Framework (https://osf.io/ct42e/?view_only=56114d585d224b5280298ea55a7e98c3/).

## Ethics Statement

The studies involving human participants were reviewed and approved by Human Subjects Committee of the Institutional Review Board at Montana State University. The patients/participants provided their written informed consent to participate in this study.

## Author Contributions

RB, MV, and MW contributed conception and design of the parent study. MH, RB, RS, and MV contributed to the aims and hypotheses of the current work. RB performed the statistical analysis. All authors wrote sections of the manuscript. All authors contributed to manuscript revision, read, and approved the submitted version.

## Conflict of Interest

The authors declare that the research was conducted in the absence of any commercial or financial relationships that could be construed as a potential conflict of interest.
